# Left Atrial Ablation of Atrial Fibrillation: Is the Fly in the Ointment a Silent Stroke?

**DOI:** 10.1111/j.1540-8167.2012.02291.x

**Published:** 2012-04-11

**Authors:** Koonlawee Nademanee, Montawatt Amnueypol

## Editorial Comment

“For never yet hath any one attained to such perfection, but that time, and place, and use, have brought addition to his knowledge; or made correction, or admonished him, that he was ignorant of much which he had thought he knew; or let him to reject what he had once esteemed of highest price.” (William Harvey, 1628, Motion of the Heart)

The above aphorism by Dr. William Harvey is an appropriate reminder to enthusiasts of catheter ablation for treatment of atrial fibrillation (AF). As witnessed over the past decade, pulmonary vein isolation (PVI), either alone or in combination with other hybrid ablative approaches (e.g., linear lesion or defragmentation), has gained popularity as an effective treatment for AF. However, with this popularity came skepticism and scrutiny of the procedure.

Critics have questioned endpoints of the procedure that define its success, and the fact that long-term 5-year success rates from the 2 leading European centers have been disappointing further fuels skepticism of the procedure.^1^,^2^ Some skeptics believe that electrophysiologists who perform the procedure have irrational exuberance for the procedure and ignore studies showing that it is not as effective as previously reported.

Critics also raise the concern that complications associated with the procedure may be underreported. They believe that in real-world clinical practice, especially in low-volume institutions, complication rates may be significantly higher than what is presented to patients. Furthermore, it remains controversial as to whether catheter ablation of AF would reduce the risk of stroke in AF patients and thus take away the need for anticoagulation.

A recent study by Gaita *et al*. showed that despite the relatively low risk of symptomatic stroke (0.4%) associated with left atrial ablation for AF, utilizing PVI or PVI plus linear lesions plus ablation of fractionated signals significantly increased the risk of asymptomatic cerebrovascular accident (CVA), as detected by magnetic resonance imaging of the brain (14%).^3^ This report unquestionably dampens the passionate enthusiasm of electrophysiologists who think that the PVI-left atrial ablation procedure should be first-line therapy for paroxysmal AF patients.

Results of this study cause several questions to be raised: How would a relatively young 58-year-old AF patient (mean age of subjects in the Gaita study was 58 years) respond to a consent form that states that the incidence of silent CVA associated with the procedure is almost 15%, and that this silent stroke may substantially increase the risk of dementia? And, undoubtedly, physicians who regularly perform left atrial ablation for AF, will ask: How can the asymptomatic stroke rate be that high, and is this rate exclusive to centers reported in Gaita's study, or can it be applied to other centers as well? What are the contributing factors to left atrial ablation for AF that cause such a high rate of stroke, and how can they be substantially reduced?

As with left heart catheterization, the left atrial ablative procedure carries a risk of thromboembolism. However, unlike the routine left heart catheterization procedure, additional contributing factors associated with catheter ablation for AF cause a greater increase in stroke risk (Table 1). The danger of thromboembolic complications starts even before the procedure begins, for AF patients who may have thrombus formation in the left atrial appendage that may cause stroke if it were missed.

**Table 1:** Variables That Could Contribute to Thromboembolic CVA Associated with Catheter Ablation of AF

• Inadequate preoperative evaluation of left atrial appendage clot
• Poor transseptal sheath management
• Perioperative anticoagulation
○ Discontinuation of anticoagulation before the procedure
○ Low-intensity heparinization during the procedure (ACT <250 seconds)
• Ablation approaches, protracted procedure time, and the need for cardioversion
• Catheter type and energy sources
○ RF energy without irrigated-tip catheter

In the Gaita study, all patients underwent a transesophageal echocardiography examination and thus it is very unlikely that thrombus formation was the factor that caused such a high incidence of silent CVA. Transseptal sheath management with careful flushing of the sheath throughout the procedure is of paramount importance, as clot could easily form in the sheath. Perioperative anticoagulation management, techniques of left atrial ablation for AF, and catheter types could also affect the stroke rate, as discussed below.

In this issue of the *Journal*, Ichiki *et al*. evaluated the incidence of CVA after complex fractionated atrial electrogram (CFAE) ablation for AF, either alone or in combination with PVI. CVA occurred in 7 out of 100 patients in this study (7%); all were asymptomatic except one.^4^ The incidence of stroke is lower in this study when compared to that of Gaita *et al*., and there are differences between these 2 studies: First, Ichiki *et al*., unlike Gaita *et al.*, did not stop oral anticoagulation for the procedure. And while both studies tried to keep activated clotting time (ACT) >250 seconds, it appears that the patients with stroke in the Ischiki study had a relatively lower ACT (mean = 274 seconds) compared with the patients with no stroke (mean = 293 seconds), albeit this did not reach statistical significance. Second, Gaita *et al*. observed that patients with ACT <250 seconds had a 17% stroke rate, whereas those with ACT >250 seconds had only a 9% stroke rate.

Next, Gaita *et al*. needed to perform cardioversion at the end of the procedure to revert AF to sinus rhythm in 27% of their patients, and they found that cardioversion is associated with an increased risk of 2.75 (95% CI) compared with those who underwent the left atrial ablation procedure during sinus rhythm or those whose AF terminated during the ablation. In contrast, Ichiki and colleagues could revert AF to sinus rhythm by catheter ablation in 95 of 100 patients (95%); only 5 patients in the persistent AF group required external cardioversion.

Whether or not the lower rate of stroke in the Ichiki study compared with the Gaita study is due to the lower percentage of patients needing external cardioversion remains unclear. What is clear, however, is that CFAE ablation, either alone or in combination with PVI, produced a high rate of AF termination by catheter ablation. This is in sharp contrast to several recently published papers, which found that the AF termination rate is quite dismal with CFAE ablation.^5^,^6^ Nevertheless, Ichiki and colleagues replicated our findings that AF ablation, by targeting CFAE, often yielded a high rate of AF termination.^7^

Over the past 12 months, we have also systematically evaluated incidence of silent stroke by brain magnetic resonance imaging before and immediately after the AF ablation procedure. Thus far, we found only one asymptomatic lacuna infarct in 1 of 70 patients (1.4%, unpublished observation). Our perioperative anticoagulation and rate of AF termination are similar to that of the Ichiki study; however, unlike Ichiki *et al*., we did not perform PVI or coronary angiography in our study—Ichiki *et al*. found that concomitant coronary angiography further increased the stroke risk in their study. Whether or not these differences in the approach result in a lower stroke rate of stroke in our experience is unknown and needs further study.

Furthermore, different catheters and energy sources employed for AF ablation greatly impact the stroke rate. Gaita and coworkers conducted another study comparing the incidence of new silent CVA in patients with PAF undergoing PVI with 1 of 3 different technologies:^8^ (1) irrigated radiofrequency catheters, (2) multielectrode catheters (PVAC) associated with duty-cycle RF generators, and (3) cryoballoon. Their findings, reported (less than 6 months ago) in this *Journal*, clearly showed that PVAC had a high and unacceptable silent CVA (39%) rate compared with irrigated RF (8.3%) and cryoballoon (5.6%).

The studies by Gaita *et al*. and Ichiki *et al*. appear to heed Dr. Harvey's guidance that we should continue to evaluate ourselves of what we do for our patients and we have learned from their studies that our approach of AF ablation is not perfect. They have brought us new knowledge that we must be concerned with asymptomatic CVA related to catheter ablation of AF, the fly in the ointment of the procedure. We must also properly manage perioperative anticoagulation, perhaps without stopping anticoagulant therapy before the AF ablative procedure and intensifying it with heparin to keep ACT above 250 seconds—preferably around 300 seconds or higher. In addition, we must abandon the use of RF energy source without an irrigated-tip catheter.

Last, we should not be content with our current approach of AF, and should search for a better way to ablate AF that is relatively simple but effective, and that does not incorporate unnecessary tools or processes, perhaps yielding a high rate of AF termination and achieving permanency of the lesion that translates into a high rate of long-term success. We are not there yet, but as enthusiasts of AF ablation, we believe that with proper correction of our techniques based on what we have learned so far and what we will learn more in the future, we will be able to provide a better outcome for our AF patients and hopefully remove the fly out of the procedure.
